# Live Poultry Exposure and Public Response to Influenza A(H7N9) in Urban and Rural China during Two Epidemic Waves in 2013-2014

**DOI:** 10.1371/journal.pone.0137831

**Published:** 2015-09-14

**Authors:** Peng Wu, Liping Wang, Benjamin J. Cowling, Jianxing Yu, Vicky J. Fang, Fu Li, Lingjia Zeng, Joseph T. Wu, Zhongjie Li, Gabriel M. Leung, Hongjie Yu

**Affiliations:** 1 WHO Collaborating Centre for Infectious Disease Epidemiology and Control, School of Public Health, Li Ka Shing Faculty of Medicine, The University of Hong Kong, Hong Kong Special Administrative Region, Hong Kong, China; 2 Division of Infectious Disease, Key Laboratory of Surveillance and Early-warning on Infectious Disease, Chinese Center for Disease Control and Prevention, Beijing, China; University of Waterloo, CANADA

## Abstract

**Background:**

The novel influenza A(H7N9) virus has caused 2013 spring and 2013–2014 winter waves of human infections since its first emergence in China in March 2013. Exposure to live poultry is a risk factor for H7N9 infection. Public psychobehavioral responses often change during progression of an epidemic.

**Methods:**

We conducted population-based surveys in southern China to examine human exposure to live poultry, and population psychological response and behavioral changes in the two waves. In Guangzhou, an urban area of Guangdong province, we collected data using telephone surveys with random digit dialing in May-June 2013 and again in December 2013 to January 2014. In Zijin county, a rural area of the same province, we used door-to-door surveys under a stratified sampling design in July 2013 and again in December 2013 to January 2014. All responses were weighted by age and sex to the respective adult populations.

**Findings:**

Around half of the urban respondents (53.8%) reported having visited LPMs in the previous year in the first survey, around double that reported in the second survey (27.7%). In the rural surveys, around half of the participants reported raising backyard poultry in the past year in the first survey, increasing to 83.2% participants in the second survey. One third of urban subjects supported the permanent closure of LPMs in the first and second surveys, and factors associated with support for closure included female sex, higher level of worry towards H7N9, and worry induced by a hypothetical influenza-like illness.

**Conclusions:**

Our study indicated high human exposure to live poultry and low support for permanent closure of markets in both urban and rural residents regardless of increased worry during the epidemic.

## Introduction

Avian influenza A(H7N9) virus has caused two major epidemics of human infections since emerging in early 2013. The first epidemic occurred in the spring of 2013 in eastern China [1, 2], while the second epidemic occurred over the winter of 2013–14 in eastern and southern China. As of 12 January 2015, there have been 490 laboratory-confirmed cases of human H7N9 infection and 188 deaths while the detection of a small number of mild cases in sentinel surveillance system implies that the true number of human infections is likely to be much greater than the reported laboratory-confirmed cases [3]. More than 80% of the confirmed H7N9 cases reported exposure to live poultry or a live poultry market (LPM) prior to symptom onset [1]. Because the H7N9 virus has low pathogenicity in poultry, unlike highly pathogenic avian influenza A(H5N1) virus, it is challenging to control the transmission of H7N9 from poultry to humans. LPMs were closed in the cities with H7N9 cases reported and/or environmental specimens testing positive for H7N9 during the spring 2013 and winter 2013–14 epidemics. Permanent closure of LPMs has been proposed in some of the cities affected by H7N9 [4, 5].

A previous study of risk factors for human infection with H5N1 suggested that patterns of poultry exposure particularly to dead/sick poultry were very different between rural and urban residents in China [6]. Our previous study suggested that population exposure to live poultry was common and risk perception to H7N9 was relatively low in China during the first wave of H7N9 epidemic in 2013 while high heterogeneity was observed in urban and rural residents across different areas [7]. Given the geographical differences observed in laboratory-confirmed cases in the two H7N9 waves, in this study we aimed to investigate patterns and changes in live poultry exposure, risk perceptions and attitudes towards H7N9, and preventive interventions adopted by the public in rural and urban areas of Guangdong province in southern China which was heavily affected during the winter 2013–14 epidemic.

## Methods

We conducted two population-based surveys in an urban area of Guangdong province (Guangzhou, capital city of the province) in southern China, and another two surveys in Zijin County, a rural area in the same province, covering both the spring 2013 epidemic and the winter 2013–14 epidemic of H7N9. Considering the feasibility and generalizability of the study, we used mobile phone surveys in Guangzhou and door-to-door surveys in Zijin [7]. We investigated human exposure to live poultry or LPMs, risk perception, psychological and behavioral responses to the outbreak, and attitudes towards the control of H7N9 through LPM closure using a structured survey instrument [7–9].

Telephone surveys were conducted using a computer-assisted telephone interviewing system, which enabled random generation of mobile telephone numbers and systematic data collection in the city. A later call was arranged if the subject was not currently available. Unanswered calls were repeated up to 4 times at different times of the week before being classified as invalid. Subjects in Zijin were selected through a stratified sampling method [7]. First, we classified towns in the county into three categories according to the gross domestic product (GDP) per capita in that town: high (3^rd^ tertile among all towns in that county), middle (2^nd^ tertile) and low (1^st^ tertile) economic levels based on the 2010 National Census, and randomly selected one town from each category. Second, two villages from each town and then 50 households from each village were randomly selected. Finally one adult who met the inclusion criteria of the study, i.e. at age of 18 years or older and living in the village for at least 1 year was selected for interview from the household. All subjects were recruited after verbal consent was obtained. In both locations, eligible participants were aged ≥18y and resident in the respective location for at least one year.

We collected data from urban and rural residents on exposure to live poultry or LPMs, level of anxiety, psychological and behavioral changes in response to the H7N9, and attitudes towards control interventions on the H7N9 epidemic. Most instruments were measured by Likert-scale responses. Measurement of exposure to live poultry in the questionnaire was different for urban and rural subjects because urban residents were mainly exposed to live poultry through visiting LPMs while backyard poultry was frequently seen in rural households and LPMs were less common in rural areas.

Demographic characteristics were analyzed for each survey and compared with chi-squared tests. Population levels of anxiety, exposure to live poultry or LPMs, perceived risk of infection with H7N9, perceived severity of H7N9, behavioral responses to H7N9, and attitudes towards LPM closure were compared between the first and second waves for urban and rural subjects weighted by age and sex of the population distribution derived from the 2010 National Census in China. Multivariate regression models were applied to investigate factors associated with the observed differences between surveys and locations in LPM visits, contact with live poultry, perceived risk of H7N9 infection, worries towards infection, preventive measures adopted and attitudes towards LPM closure as a effective intervention to control H7N9 epidemics. Statistical analyses were performed in R version 3.0.2.

### Ethics Statement

Ethical approval for this study was obtained from the Institutional Review Board (IRB) of the Chinese Center for Disease Control and Prevention. The IRB approved that verbal consent should be obtained from all participants. Subjects from whom consent was obtained were asked to complete the questionnaire.

## Results

Two surveys in Guangzhou were conducted in May-June 2013 (Survey U1), and between December 2013 and January 2014 (Survey U2), with 500 and 549 interviews completed, respectively. Two surveys in the rural area of Zijin County recruited 308 subjects in July 2013 (Survey R1), and 300 between December 2013 and January 2014 (Survey R2). More young adults were recruited in the urban than rural area in all surveys. Urban subjects reported having relatively higher educational level on average in Survey U1 than those in Survey U2 (S1 Table).

Around half of the urban respondents (53.8%) reported having visited LPMs in the previous year in the first survey, around double that reported in the second survey (27.7%). Urban respondents in the first survey reported purchasing an average of 36.5 live poultry in the past year, while respondents in the second survey reported an average of 26.7 live poultry purchased in the past year. Of those who reported buying live poultry at least once, the proportion of respondents who would always touch live poultry declined considerably from 50.2% in the first survey to 20.6% in the second survey. In the rural surveys, around half of the subjects reported raising backyard poultry in the past year in the first survey, and this increased to 83.2% in the second survey. Around 70%-80% urban subjects in both surveys reported buying less live poultry since H7N9 cases were detected in China. During the second survey, 58.5% of rural subjects reported that they changed live poultry purchase behavior since the H7N9 virus reemerged in winter 2013–14. One third of urban subjects supported the permanent closure of LPMs in the first and second surveys while only 11% of rural subjects supported LPM closure in the second survey.

Urban subjects showed a higher general anxiety and more worry about H7N9 in the second than the first survey while rural subjects had slightly lower general anxiety score (1.74 vs 1.86) but greater worry about H7N9 (5.71 vs 4.20) in the second survey (Table 1). The proportion of urban subjects who perceived a higher absolute susceptibility to H7N9 (2.6%) and higher relative susceptibility (0.7%) in the first survey is lower than that reported in the second survey, 4.3% and 3.8% for absolute and relative susceptibility, respectively. In the rural area, few subjects perceived higher absolute and relative susceptibility to H7N9 in the first survey while a considerably increased proportion of subjects perceiving higher absolute (16.3%) and relative (10.4%) susceptibility was observed in the second survey. In general, both urban and rural subjects had a higher ILI symptom-induced worry against H7N9 in the second than the first survey.

**Table 1:** Risk perception to H7N9 in urban and rural subjects recruited in Guangdong province during the two surveys in 2013–14.

	Urban			Rural		
	Survey 1 (N = 500), n (%)	Survey 2 (N = 549), n (%)	p value	Survey 1 (N = 308), n (%)	Survey 2 (N = 300), n (%)	p value
**Mean STAI scores**	1.8	2.0	<0.01	1.9	1.7	<0.01
**Worry about H7N9** ^1^	3.8	4.8	<0.01	4.2	5.7	<0.01
**Perceived absolutesusceptibility** ^2^			0.07			<0.01
High	9 (2.6)	23 (4.3)		1 (0.3)	45 (16.3)	
Even	98 (21.1)	99 (18.1)		41 (13.0)	80 (25.2)	
Low	393 (76.4)	427 (77.6)		266 (86.7)	175 (58.4)	
**Perceived relative susceptibility** ^3^			<0.01			<0.01
High	5 (0.7)	18 (3.8)		1 (0.3)	28 (10.4)	
Even	52 (11.2)	37 (6.1)		25 (7.1)	93 (32.9)	
Low	443 (88.2)	494 (90.1)		282 (92.6)	179 (56.7)	
**ILI symptoms induced worry** ^4^			<0.01			<0.01
More	151 (29.8)	244 (41.0)		79 (25.0)	150 (52.6)	
Same as usual	198 (41.0)	170 (32.2)		113 (34.9)	77 (25.0)	
Less	151 (29.2)	135 (26.8)		116 (40.1)	73 (22.4)	
**Infection with H7N9 in the past week** ^5^			<0.01			<0.01
Worry	68 (14.2)	139 (23.7)		76 (25.4)	144 (50.4)	
Think about it but no worry	57 (9.9)	135 (24.0)		42 (12.9)	40 (12.0)	
Never think about it	375 (75.9)	275 (52.3)		190 (61.6)	116 (37.6)	
**Relative severity of H7N9** ^6^						
Compared to seasonal flu	319 (65.6)	304 (51.9)	<0.01	181 (56.8)	204 (71.9)	0.02
Compared to H5N1 avian flu	163 (32.5)	237 (42.5)	<0.01	112 (34.1)	163 (60.2)	<0.01
Compared to SARS	57 (10.8)	113 (21.4)	<0.01	63 (19.4)	34 (10.8)	<0.01
**Knowledge towards H7N9 transmission**						
Contact poultry in LPMs	404 (80.8)	382 (69.1)	<0.01	232 (73.1)	247 (84.3)	0.88
Contact H7N9 patients	261 (51.6)	332 (60.3)	0.01	198 (62.1)	278 (92.4)	<0.01
Contact virus-contaminated objects	378 (77.3)	408 (74.4)	0.75	225 (70.9)	292 (97.2)	<0.01

^1^: Subjects were asked to rate the worry about H7N9 with a number in 1–10.

^2^: Subjects who answered certain/very likely/likely to the question “How likely do you think it is that you will contract H7N9 avian flu over the next 1 month?” were categorized as “High” in the table while those who answered never/very unlikely/unlikely were categorized as “Low”.

^3^: Subjects who answered certain/much more /more to the question “What do you think is your chance of getting infected with H7N9 avian flu over the next 1 month compared to other people outside your family of a similar age?” were categorized as “High” in the table while those who answered not at all/much less/less were categorized as “Low”.

^4^: Subjects who answered extremely concerned/concerned much more than normal/concerned more than normal to the question “If you were to develop ILI symptoms tomorrow, would you be…?” were categorized as “More” in the table while those who answered not at all concerned/much less concerned than normal/ concerned less than normal were categorized as “Less”.

^5^: Subjects who answered worried about it all the time/worried a lot/worried a bit to the question “Did you worry about H7N9 in the past week?”were categorized as “Worry” in the table.

^6^: Subjects who answered much higher/a little higher regarding the severity of H7N9 compared to seasonal influenza, H5N1 avian influenza and SARS were used to the numbers and proportions in the table. Proportions in the table have been weighted by age and sex to the population distribution in the National Census 2010.

Abbreviations: SARS, severe acute respiratory syndrome; ILI, influenza-like illness.

We examined factors affecting poultry purchase behaviors and support of LPM closure (Table 2). We found that married subjects and those having received higher level of education were likely to have lower exposure to live poultry after adjusting for other confounding factors. Touching poultry during purchase in LPMs also declined in the second survey (odds ratio (OR): 0.3, 95% confidence interval (CI): 0.2, 0.6), and those worrying more about H7N9 were likely to touch less. Female sex, older age, and those having higher level of worry, perceiving higher severity or knowing that H7N9 can be transmitted by contact were associated with changes in poultry purchase behaviors (Table 2), while support for the permanent closure of LPMs was only associated with female sex, higher level of worry towards H7N9 and ILI-induced worry, and marginally associated with the perceived effectiveness of H7N9 control by the government.

**Table 2:** Factors associated with poultry exposure and attitudes and behavior towards H7N9 in subjects recruited in urban area of Guangdong province during the two surveys in 2013–14.

	Frequency of LPM visits Relative risk (95% CI)	Frequency of poultry purchase Relative risk (95% CI)	Touching poultry during purchase Odds ratio (95% CI)	Support closure of LPMs Odds ratio (95% CI)	Change purchase behavior Odds ratio (95% CI)
**Survey**					
Survey 1	Reference	Reference	Reference	Reference	Reference
Survey 2	**-22.5 (-34.2, -10.8)**	**-12.2 (-22.3, -2.1)**	**0.3 (0.2, 0.6)**	1.0 (0.7, 1.6)	1.1 (0.7, 1.6)
**Gender**					
Male	Reference	Reference	Reference	Reference	Reference
Female	0.7 (-10.1, 11.5)	-0.5 (-9.8, 8.7)	0.8 (0.5, 1.2)	**1.7 (1.2, 2.4)**	**1.7 (1.1, 2.3)**
**Age group (years)**					
18–24	Reference	Reference	Reference	Reference	Reference
25–34	12.3 (-3.0, 27.6)	5.9 (-7.6, 19.4)	**0.4 (0.2, 0.8)**	0.8 (0.5, 1.3)	**2.5 (1.5, 4.1)**
35–54	6.3 (-14.1, 26.6)	3.4 (-13.7, 20.4)	**0.3 (0.1, 0.7)**	0.7 (0.4, 1.4)	**2.3 (1.2, 4.4)**
≥55	-0.3 (-21.8, 21.2)	3.7 (-15.5, 23.0)	**0.4 (0.1, 0.9)**	1.0 (0.5, 2.0)	**3.0 (1.5, 6.3)**
**Marital status**					
Married/previously married	Reference	Reference	Reference	Reference	Reference
Single	**-17.2 (-30.4, -3.9)**	**-11.3 (-22.2, -0.4)**	0.8 (0.5, 1.4)	0.9 (0.6, 1.3)	0.8 (0.5, 1.3)
**Educational attainment**					
Primary or below	Reference	Reference	Reference	Reference	Reference
Secondary	4.5 (-17.8, 26.8)	-19.6 (-41.2, 2.1)	0.9 (0.4, 2.4)	2.0 (0.9, 4.8)	1.0 (0.5, 2.4)
Tertiary or above	1.2 (-21.6, 24.1)	**-25.6 (-47.3, -3.9)**	0.7 (0.3, 1.9)	1.8 (0.8, 4.3)	1.5 (0.6, 3.4)
**Anxiety level (STAI Score)**					
1^st^ tertile	Reference	Reference	Reference	Reference	Reference
2^nd^ tertile	-0.7 (-13.2, 11.7)	-9.3 (-20.2, 1.7)	0.9 (0.5, 1.5)	1.2 (0.8, 1.8)	1.2 (0.8, 1.9)
3^rd^ tertile	**16.0 (2.1, 29.8)**	-7.4 (-19.2, 4.4)	0.7 (0.4, 1.2)	1.4 (0.9, 2.2)	1.1 (0.7, 1.7)
**Worry about H7N9**					
1^st^ tertile	Reference	Reference	Reference	Reference	Reference
2^nd^ tertile	3.0 (-9.9, 15.9)	-11.1 (-22.3, 0.2)	**0.6 (0.3, 1.0)**	1.2 (0.8, 1.8)	**1.7 (1.1, 2.7)**
3^rd^ tertile	0.5 (-12.8, 13.7)	-10.1 (-21.5, 1.2)	**0.5 (0.3, 0.9)**	**2.0 (1.3, 2.9)**	**1.9 (1.2, 3.0)**
**Perceived self susceptibility**					
Low	Reference	Reference	Reference	Reference	Reference
High	-5.7 (-37.0, 25.7)	10.2 (-14.7, 35.2)	2.6 (0.8, 8.5)	1.1 (0.5, 2.7)	0.7 (0.3, 1.7)
**Perceived relative susceptibility**					
Low	Reference	Reference	Reference	Reference	Reference
High	-7.2 (-43.5, 29.1)	17.5 (-10.1, 45.1)	0.3 (0.1, 1.5)	1.2 (0.5, 3.2)	1.1 (0.4, 3.4)
**ILI induced worry**					
Low	Reference	Reference	Reference	Reference	Reference
High	7.4 (-3.8, 18.6)	8.2 (-1.1, 17.5)	1.4 (0.9, 2.2)	**1.7 (1.2 2.4)**	**1.9 (1.3, 2.8)**
**Perceived relative severity**					
**Compared with seasonal influenza**					
Low	Reference	Reference	Reference	Reference	Reference
High	1.1 (-10.3, 12.6))	-5.7 (-15.6, 4.2)	0.9 (0.6, 1.5)	0.9 (0.6, 1.3)	**1.6 (1.1, 2.4)**
**Compared with H5N1 influenza**					
Low	Reference	Reference	Reference	Reference	Reference
High	6.4 (-5.5, 18.2)	-2.0 (-12.0, 8.1)	1.2 (0.7, 1.9)	1.4 (0.9, 2.0)	1.0 (0.7, 1.5)
**Compared with SARS**					
Low	Reference	Reference	Reference	Reference	Reference
High	8.7 (-6.0, 23.3)	8.8 (-3.3, 20.8)	1.2 (0.6, 2.1)	1.3 (0.8, 1.9)	0.9 (0.6, 1.4)
**Perceived effectiveness of H7N9 control**					
National government	-0.1 (-2.9, 2.8)	-2.2 (-2.6, 0.1)	**1.1 (1.0, 1.2)**	**1.1 (1.0, 1.2)**	1.0 (0.9, 1.1)
Local government	0.1 (-2.6, 2.9)	0.5 (-1.9, 2.8)	**1.1 (1.0, 1.2)**	**1.1 (1.0, 1.2)**	1.0 (0.9, 1.1)
**Knowledge about H7N9 transmission**					
Contract poultry in LPMs	-1.4 (-14.3, 11.4)	-0.8 (-11.7, 10.0)	1.0 (0.6, 1.8)	1.1 (0.7, 1.7)	**1.6 (1.1, 2.4)**
Contact H7N9 patients	-5.7 (-17.1, 5.7)	-7.9 (-17.5, 1.6)	**0.6 (0.4, 1.0)**	0.9 (0.6, 1.3)	1.3 (0.9, 1.9)
Contact virus-contaminated objects	0.6 (-12.5, 13.6)	-9.2 (-20.3, 1.9)	1.2 (0.7, 2.0)	1.4 (0.9, 2.2)	0.9 (0.6, 1.4)

In rural respondents, we examined factors affecting backyard poultry, support for LPM closure and changes in purchasing behaviors (S2 Table). We found that respondents were more likely to support closure of LPMs if they were female, had greater worry about H7N9, higher perceived susceptibility, greater ILI symptom-induced worry, or lower perceived severity of H7N9 relative to H5N1. Rural respondents were more likely to report a change in their purchasing behavior if they were more educated, had greater ILI symptom-induced worry, perceived greater severity compared to seasonal influenza or H5N1, knowing that H7N9 can be transmitted by contact, or perceived less effectiveness of H7N9 control by the government.

## Discussion

**Fig 1:**
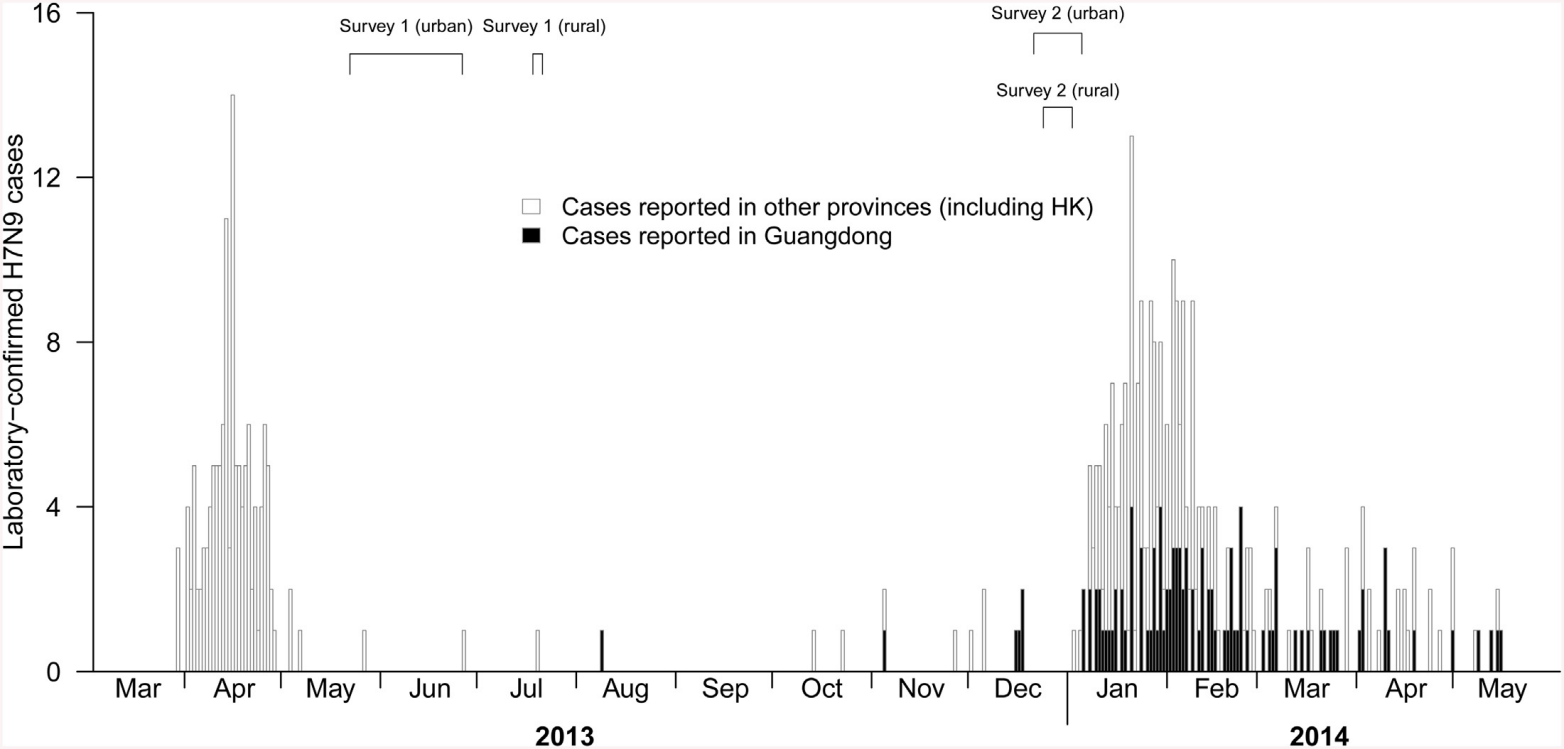
Laboratory-confirmed influenza A(H7N9) by date of reporting in Guangdong (black bars) and other provinces in China (white bars) from March 2013 through May 2014 and the time of conducting surveys in the urban and rural areas in Guangdong province.

The impact of two epidemics caused by H7N9 virus was different in mainland China during spring 2013 and winter 2013–14 epidemics with more laboratory-confirmed cases across a broader geographical area in the second wave. Human infection with H7N9 was associated with exposure to live poultry or LPMs from epidemiological investigations on laboratory-confirmed H7N9 cases for risk factors during the first wave in 2013 [1, 2, 10, 11]. A small number of H7N9 cases were reported in the spring 2013 epidemic in Guangdong province where our surveys were conducted while more cases were reported during the second epidemic in winter 2013–14 (Fig 1), indicating higher transmission of H7N9 during that period. The declining exposure to live poultry and LPMs in urban subjects in the second wave compared to the first wave was consistent with greater worry among respondents (Table 1), leading to changes in protective behaviors such as less visits to LPMs and less purchase of live poultry (Table 2) while there was a seasonal increase in poultry demand due to the Spring Festival in both urban and rural China from the late January to the early February. Nevertheless, exposure to live poultry was still common in both epidemics.

No apparent reduction was reported by the rural subjects in terms of backyard poultry exposure in this study, suggesting that rural subjects were probably aware that most detected H7N9 cases exposed to urban LPMs/live poultry instead of backyard poultry, or that factors associated with live poultry exposure in the rural area might be different from those in urban cities. The inconsistency between the higher level of worries and perceived risk of infection and the unchanged poultry exposure in rural subjects implied that decision-making on raising backyard poultry might not be driven by these factors although contact with live poultry or virus-contaminated objects was regarded as the possible routes of H7N9 transmission by most of rural subjects (Table 1). Changes in raising backyard poultry in the rural area was different from patterns observed in the urban area on live poultry exposure in both waves possibly implying that circulation of H7N9 virus in rural backyard poultry was less than that detected in urban LPMs.

LPM closure has been suggested highly effective in reducing H7N9 transmission in the H7N9 epidemic [12]. Reduction of live poultry exposure reported by urban subjects in two surveys to some extent reflected the effect of market closure although poultry demands usually increase during the Chinese Lunar New Year and the change in live poultry contact could also be due to increased risk perception to the virus in the public or seasonal variation in poultry exposure. Around one third of urban respondents supported closure of LPMs in both surveys (Table 1) despite much more proximal risk in the second survey, during the winter 2013–14 epidemic when many cases were reported in Guangzhou. Perceived susceptibility was not a significant predictor of support for LPM closure (Table 2).

There are a number of limitations to our study. As with all observational studies, our findings may have been affected by reporting biases such as recall bias or social desirability bias, the latter occurring if respondents provided answers that they considered to be more desirable than the truth. Because we used telephone surveys in urban areas and face-to-face interviews in rural areas, and responses may differ depending on the mode of data collection, we avoided direct quantitative comparisons between the two settings. Our present study focused on an urban and rural area in southern China, and the findings may not generalize to other parts of China.

In conclusion, live poultry exposures were common in urban and rural areas of Guangdong province. Exposures declined in urban residents during winter 2013–14, at least partly due to the H7N9 epidemic. Changes in poultry purchasing behavior were associated with greater worry about H7N9. A minority of urban respondents supported permanent LPM closure. Further efforts are needed to protect public health if H7N9 re-emerges in a third epidemic in the winter of 2014–15.

## Supporting Information

S1 Table Socio-demographic characteristics of subjects recruited in the urban and rural areas of Guangdong province during the two surveys in 2013–14. (DOCX)

S2 Table Factors associated with poultry exposure and attitudes and behavior towards H7N9 in subjects recruited in rural area of Guangdong province during the two surveys in 2013–14. (DOCX)
