# Supplementary material for: Live Poultry Exposure and Public Response to Influenza A(H7N9) in Urban and Rural China during Two Epidemic Waves in 2013-2014
Source: PLoS One. 2015 Sep 14;10(9):e0137831. doi: 10.1371/journal.pone.0137831 (PMC4569561; doi:10.1371/journal.pone.0137831)
Supplement: S1 Table — (DOCX) [file pone.0137831.s001.docx]

Table S1. Socio-demographic characteristics of subjects recruited in the urban and rural areas of Guangdong province during the two surveys in 2013-14.

|  | **Urban (%)** | | | **Rural (%)** | | |
| --- | --- | --- | --- | --- | --- | --- |
|  | **Survey 1**  **(n=500)** | **Survey 2**  **(n=549)** | **p value** | **Survey 1**  **(n=308)** | **Survey 2 (n=300)** | **p value** |
| **Gender** |  |  |  |  |  |  |
| Male | 52.6 | 49.0 | 0.27 | 47.7 | 51.7 | 0.37 |
| **Age group (years)** |  |  | <0.01 |  |  | <0.01 |
| 18-24 | 21.8 | 19.7 |  | 22.7 | 35.3 |  |
| 25-34 | 40.8 | 50.8 |  | 25.0 | 11.0 |  |
| 35-54 | 21.8 | 13.5 |  | 29.0 | 21.7 |  |
| ≥55 | 15.6 | 16.0 |  | 22.7 | 32.0 |  |
| **Educational attainment^1^** |  |  | <0.01 |  |  | <0.01 |
| Primary | 10.4 | 5.7 |  | 20.5 | 21.9 |  |
| Secondary | 44.9 | 27.4 |  | 67.7 | 73.3 |  |
| Tertiary or above | 44.7 | 66.9 |  | 11.8 | 4.8 |  |
| **Marital status^1^** |  |  | 0.21 |  |  | 0.70 |
| Single | 34.6 | 38.7 |  | 16.5 | 15.0 |  |
| Married/previously married | 65.5 | 61.9 |  | 83.6 | 84.9 |  |
| **Monthly household income (CNY^2^)^1^** |  |  | <0.01 |  |  | <0.01 |
| < 3000 | 15.8 | 18.8 |  | 60.3 | 16.6 |  |
| 3001-6000 | 21.5 | 36.7 |  | 19.4 | 73.8 |  |
| 6001-10,000 | 16.1 | 20.3 |  | 9.1 | 7.4 |  |
| > 10000 | 20.5 | 24.2 |  | 3.8 | 2.2 |  |
| Not reported | 26.1 | 0.0 |  | 7.4 | 0.0 |  |

^1^Proportions for educational attainment, marital status and monthly household income have been weighted by age and sex to the population distribution obtained in the 2010 National Census.

^2^ CNY 6.1 = USD 1
