# Supplementary material for: Live Poultry Exposure and Public Response to Influenza A(H7N9) in Urban and Rural China during Two Epidemic Waves in 2013-2014
Source: PLoS One. 2015 Sep 14;10(9):e0137831. doi: 10.1371/journal.pone.0137831 (PMC4569561; doi:10.1371/journal.pone.0137831)
Supplement: S2 Table — (DOCX) [file pone.0137831.s002.docx]

Table S2. Factors associated with poultry exposure and attitudes and behavior towards H7N9 in subjects recruited in rural area of Guangdong province during the two surveys in 2013-14.

|  | **Raised Backyard poultry**  **Odds ratio (95% CI)** | **No. of backyard poultry raised**  **Relative risk (95% CI)** | **Support closure of LPMs**  **Odds ratio (95% CI)** | **Change purchase behavior**  **Odds ratio (95% CI)** |
| --- | --- | --- | --- | --- |
| **Survey** |  |  |  |  |
| Survey 1 | Reference | Reference | - | - |
| Survey 2 | **5.5 (3.4, 9.0)** | -3.0 (-7.3, 1.2) | - | - |
| **Gender** |  |  |  |  |
| Male | Reference | Reference | Reference | Reference |
| Female | 1.0 (0.7, 1.5) | **-4.4 (-7.5, -1.2)** | **0.2 (0.1, 0.6)** | 0.6 (0.3, 1.3) |
| **Age group (years)** |  |  |  |  |
| 18-24 | Reference | Reference | Reference | Reference |
| 25-34 | 1.1 (0.5, 2.4) | -3.4 (-10.9, 4.1) | 0.5 (0.1, 4.3) | **0.2 (0.03, 0.8)** |
| 35-54 | **3.5 (1.6, 7.7)** | 0.9 (-6.4, 8.1) | 0.4 (0.1, 3.2) | 0.3 (0.1, 1.3) |
| ≥55 | 1.9 (0.8, 4.3) | 1.2 (-6.7, 9.0) | 0.8 (0.1, 7.5) | 0.8 (0.2, 4.1) |
| **Marital status** |  |  |  |  |
| Married/previously married | Reference | Reference | Reference | Reference |
| Single | 1.0 (0.5, 2.1) | -3.0 (-9.9, 4.0) | 0.7 (0.11, 4.8) | 0.3 (0.1, 1.1) |
| **Educational attainment** |  |  |  |  |
| Primary or below | Reference | Reference | Reference | Reference |
| Secondary | 1.5 (0.8, 2.6) | -1.0 (-5.7, 3.8) | 1.7 (0.4, 7.5) | 2.0 (0.6, 6.6) |
| Tertiary or above | 0.9 (0.4, 2.1) | 1.1 (-6.7, 8.9) | 5.2 (0.7, 39.5) | **6.9 (1.1, 42.2)** |
| **Anxiety level (STAI Score)** |  |  |  |  |
| 1^st^ tertile | Reference | Reference | Reference | Reference |
| 2^nd^ tertile | 1.3 (0.8, 2.1) | 2.6 (-1.5, 6.6) | 2.4 (0.9, 6.8) | 0.6 (0.2, 1.4) |
| 3^rd^ tertile | 1.3 (0.8, 2.1) | 0.4 (-3.9, 4.7) | 0.9 (0.3, 3.1) | 0.6 (0.2, 1.7) |
| **Worry about H7N9** |  |  |  |  |
| 1^st^ tertile | Reference | Reference | Reference | Reference |
| 2^nd^ tertile | 1.0 (0.6, 1.6) | 2.2 (-2.0, 6.5) | **8.1 (1.0, 66.5)** | 0.6 (0.3, 1.7) |
| 3^rd^ tertile | 0.9 (0.5, 1.4) | **5.8 (1.3, 10.3)** | **21.7 (2.6, 179.9)** | 2.6 (0.9, 7.3) |
| **Perceived absolute susceptibility** |  |  |  |  |
| Low | Reference | Reference | Reference | Reference |
| High | 0.9 (0.3, 2.9) | -0.7 (-7.3, 5.9) | **3.6 (1.1, 11.8)** | ^^ |
| **Perceived relative susceptibility** |  |  |  |  |
| Low | Reference | Reference | Reference | Reference |
| High | 7.4 (0.8, 66.0) | 1.4 (-6.0, 8.8) | 1.4 (0.4, 4.8) | 1.3 (0.1, 13.8) |
| **ILI induced worry** |  |  |  |  |
| Low | Reference | Reference | Reference | Reference |
| High | **1.7 (1.1, 2.8)** | 3.6 (-0.1, 7.3) | **1.5 (0.6, 4.1)** | **6.9 (3.3, 14.7)** |
| **Perceived relative severity** |  |  |  |  |
| **Compared with seasonal influenza** |  |  |  |  |
| Low | Reference | Reference | Reference | Reference |
| High | 1.3 (0.8, 1.9) | 1.1 (-3.0, 5.2) | 0.9 (0.3, 3.0) | **3.2 (1.3, 7.9)** |
| **Compared with H5N1 influenza** |  |  |  |  |
| Low | Reference | Reference | Reference | Reference |
| High | **1.8 (1.2, 2.8)** | 1.2 (-2.7, 5.1) | **0.3 (0.1, 0.9)** | **2.4 (1.0, 5.6)** |
| **Compared with SARS** |  |  |  |  |
| Low | Reference | Reference | Reference | Reference |
| High | 1.5 (0.9, 2.6) | -1.6 (-6.1, 2.9) | 3.2 (0.9, 11.4) | 0.9 (0.3, 2.5) |
| **Perceived effectiveness of H7N9 control** |  |  |  |  |
| National government | **1.1 (1.0, 1.3)** | -0.6 (-1.6, 0.5) | 1.0 (0.6, 1.7) | 1.1 (0.8, 1.6) |
| Local government | **0.9 (0.8, 1.0)** | 0.3 (-0.7, 1.3) | 0.8 (0.4, 1.3) | **0.6 (0.4, 0.8)** |
| **Knowledge about H7N9 transmission** |  |  |  |  |
| Contract poultry in LPMs | **0.6 (0.4, 1.0)** | -0.8 (-5.0, 3.3) | 0.3 (0.1, 1.3) | **3.2 (1.2, 8.1)** |
| Contact H7N9 patients | 0.7 (0.4, 1.2) | 1.1 (-4.1, 6.3) | 1.3 (0.1, 12.9) | 0.4 (0.1, 1.3) |
| Contact virus-contaminated objects | 1.0 (0.5, 1.8) | -4.4 (-10.6, 1.8) | ^^ | 0.9 (0.1, 6.8) |

^^ Very high odds ratios were estimated due to: (1) Subjects who agreed that H7N9 can transmit through contact with virus-contaminated objects all supported permanent closure of live poultry markets; (2) Subjects who perceived high absolute susceptibility all changed their live poultry purchase behavior since H7N9 was detected
